# The Clinical Application of 3D-Printed Boluses in Superficial Tumor Radiotherapy

**DOI:** 10.3389/fonc.2021.698773

**Published:** 2021-08-19

**Authors:** Xiran Wang, Xuetao Wang, Zhongzheng Xiang, Yuanyuan Zeng, Fang Liu, Bianfei Shao, Tao He, Jiachun Ma, Siting Yu, Lei Liu

**Affiliations:** ^1^Department of Head and Neck Oncology, West China Hospital, Sichuan University, Chengdu, China; ^2^Department of Radiotherapy, West China Hospital, Sichuan University, Chengdu, China

**Keywords:** superficial tumor, radiotherapy, radiation dermatitis, cost-effectiveness, three-dimensional bolus

## Abstract

During the procedure of radiotherapy for superficial tumors, the key to treatment is to ensure that the skin surface receives an adequate radiation dose. However, due to the presence of the built-up effect of high-energy rays, equivalent tissue compensators (boluses) with appropriate thickness should be placed on the skin surface to increase the target radiation dose. Traditional boluses do not usually fit the skin perfectly. Wet gauze is variable in thickness day to day which results in air gaps between the skin and the bolus. These unwanted but avoidable air gaps lead to a decrease of the radiation dose in the target area and can have a poor effect on the outcome. Three-dimensional (3D) printing, a new rising technology named “additive manufacturing” (AM), could create physical models with specific shapes from digital information by using special materials. It has been favored in many fields because of its advantages, including less waste, low-cost, and individualized design. It is not an exception in the field of radiotherapy, personalized boluses made through 3D printing technology also make up for a number of shortcomings of the traditional commercial bolus. Therefore, an increasing number of researchers have tried to use 3D-printed boluses for clinical applications rather than commercial boluses. Here, we review the 3D-printed bolus’s material selection and production process, its clinical applications, and potential radioactive dermatitis. Finally, we discuss some of the challenges that still need to be addressed with the 3D-printed boluses.

## Introduction

The maximum radiation dose of high-energy external X-ray beams can be reached only after they enter the human tissue with a certain depth, which is known as the built-up effect or skin-sparing effect (1–3). This effect can protect the skin during radiotherapy for deep-seated tumors, but for cancers located close to the skin (like malignant melanoma, head-and-neck cancer, postmastectomy radiotherapy, etc.), which may reduce the target coverage dose, result in the poor therapeutic efficacy of radiotherapy (4–6). Therefore, in terms of radiotherapy for superficial tumors, tissue-equivalent boluses need to be placed on the skin’s surface to reduce the risk of local recurrence and improve the long-term survival rate (7, 8). But perfect contact with skin with the current bolus is difficult, air gaps between the bolus and skin lead to an inadequate or inhomogeneous radiation dose to the surface of the skin (9, 10).

The emerging 3D printing technology known as “additive manufacturing” creates 3D structures through successive layers of material from the bottom up (11, 12), instead of cutting or milling out the shape of the object from a larger volume of material, or casting molten material in a mold. This technology has widespread application in all walks of life because of its low-cost and ease of use. There is great opportunity for 3D printing development in medicine, and the abundance of new research is showing the ability of 3D printing to promote innovation (13–15).

3D printing technology is also of enormous clinical value in the field of radiation oncology, whether in quality-assured phantoms and brachytherapy applications or in beam modulators and boluses (16–19). The 3D-printed bolus is one of many research advances in 3D printing technology, broadening its prospects (10, 20, 21). In comparison with the traditional bolus, individualized 3D-printed boluses have many advantages: Firstly, the more uniform thickness reduces ray scattering and avoids hot and cold spots (20, 22–24). Secondly, the area covered by the 3D-printed bolus is more accurate; reducing unnecessary dose increases to distant organs. Furthermore, modern radiotherapy techniques, like modulation of electron radiation therapy (MERT), achieve technically accurate control of the shape of the bolus and improve the parameters of dosimetry, thus attaining better delivery of therapeutic dose and protecting the organs at risk (OARs) (25–30).

In this article, we will give an overview of the application of the 3D-printed bolus in radiotherapy. Preclinical studies focused on the selection of materials and fabrication of 3D-printed boluses, and preliminary dosimetry validation on the human body model. Then the clinical research explored dosimetry, skin side effects, and cost-effectiveness.

## Fabrication of the 3D-Printed Bolus

### Bolus Materials

Bolus-assisted radiotherapy can effectively control the local recurrence of cancers. The key point is to make sure the bolus has perfect contact with skin. Taking into account the physical characteristics, biocompatibility, safety of the material, and filling ratio of the bolus, as well as the comfort of the finished product under different manufacturing processes is necessary (23, 26, 31, 32).

The most commonly used materials are polylactic acid (PLA) and acrylonitrile butadiene styrene (ABS) with a high filling ratio (20, 29, 33). The densities of both materials are similar with water (1-1.2 g/cm^3^), the printing setting temperature of ABS is slightly higher than PLA (25, 34, 35). Sarah Burleson and Kwangwoo Park et al. pointed out that PLA (Shaw’s hardness 75D) was closer to soft tissue than ABS (Shaw’s hardness 70D), improving the anatomical compliance, and reducing the problem of bending or warping during printing (30, 34). But these two materials and their modifications (ABS-M30, ABS plus, polyamide PA2200) are extremely hard and cannot be used as boluses for patients with sensitive skin or open wounds; new materials have been explored in recent years (31, 36–38). Clear Flex 30 (urethane liquid rubber) has been reported to irritate the eyes, respiratory system, and skin, and may cause sensitization through inhalation and skin contact (26). Agilus-60 is a soft‐curing rubber‐like photopolymer resin, which remains soft and semi‐flexible, but this material is rare and expensive (23). Yi Hou et al. explored a new bolus material-hydrogel, although with good conformability and adhesion and antibacterial effect, its practicality still needs to be further studied due to its complicated production process and the fact that only pre-clinical studies have been undertaken (32). Some studies have shown that silicon exhibited excellent physical properties in terms of flexibility (density 1.131g/C, shore hardness 10A), durability [tensile strength of 475 psi and elongation at break of 1000 (%)], without cytotoxicity and skin irritation (26, 39, 40) (details are shown in **Table 1**).

**Table 1:** Different materials of the 3D-printed bolus.

Authors	Lesion	Material	Characteristic
Su et al. (33)	Head	PLA	CT value:160 ± 20 HU. Density: 1.119 ± 0.012 g/cm^3.^
Canters et al. (20)	Skin	PLA	Printing temperature: 220°C. Layer thickness: 0.3 mm (20% infill).
So-Yeon Park (29)	Breast	PLA	Thickness: 3-5 mm. CT value: 274 HU. Fill density: 1 g/cm3. Extruder temperature: 240°C.
Park et al. (30)	Breast	PLA	Thickness: 2 mm. Density: 1.19 g/cm^3^. Layer thickness: 0.5 mm (100% infill).
Robar et al. (25)	Breast	PLA	Thickness: 5 mm. Density: 1.1 to 1.2 (100% infill). Electron density: 3.8x10^23^ electrons/cm^3^.
Burleson et al. (34)	Nose	PLA, ABS	Density: 1.2 g/cm^3^, 1.04 g/cm^3^. Electron density: 1.14, 1.01. Hydrogen content: 6%, 8%. Effective Z: 4.22, 3.45.
Ricotti et al. (35)	RANDO phantom	PLA, ABS	Density: 1.2 g/cm^3^, 1.04 g/cm^3^. Extruder temperature: 180°C, 210°C.
Craft et al. (31)	RANDO phantom	PLA, ABS, NinjaFlex, Cheetah	CT value: 30 HU (ABS, Cheetah), 121 HU (PLA), 178 HU (NinjaFlex). Density: 0.97, 1.18, 1.07, 1.15.
Zou et al. (41)	RANDO phantom	PLA, polyamide PA2200	CT value: 130.1 ± 10.1, -72.1 ± 5.3 HU. Density: 1.19 ± 0.03 g/cm^3^, 0.97 ± 0.02 g/cm^3^. Proton stopping power: 1.10, 0.98. Technology: FDM and SLS
Zhao et al. (38)	Head	PLA and NinjaFlex	Thickness: 10 mm. Layer deposition height: 0.3 mm (100% infill).
Zhang et al. (42)	Phantom	ABS	CT value is 38 ~ 73 HU. Electron density: 1.00 ~ 1.01
Kim et al. (36)	Nose	ABS-M30	Thickness: 1 cm. CT value: -123.6 ± 18.2 HU Density: 1.04 g/cm^3^
Park et al. (37)	Auricle	ABSplus	Layer thickness: 0.254 mm.
Park et al. (26)	Head	Silicon, Clear Flex 30	Thickness: 1 cm. Average HU: 161, -7. Mass densities (g/cc): 1.131, 0.998. Shore hardness: 10A, 30A. Extruder temperature: 245°C.
Chiu (40)	Head	Silicon	Outer shell thickness: 2 mm. CT value: 139.5 ± 6.4 HU. Density: 1.07 g/cm3. Layer height: 0.3 mm (3% infill).
Park et al. (39)	Nose	Rubber-like	Thickness: 3-5 mm. Technology: PolyJet.
Baltz et al. (23)	Head	Agilus-60	Thickness: 5 mm. CT value: 84 ± 33 HU. Density: 1.09 g/cm^3^.
Hou et al. (32)	Head	TPU-PAAm hydrogel	HU value and PDD: Same as H_2_O

PLA, polylactic acid; ABS, acrylonitrile butadiene styrene; TPU-PAA hydrogel, incorporated polyurethane/polyacrylamide hydrogel; CT, computed tomography; HU, Hounsfield units; FDM, fused deposition modeling; SLS, selective laser sintering; PDD, percent depth dose.

As we can see, there are many different materials for 3D-printed boluses and without a uniform standard. The bolus substance must be odorless, non-sticky, and harmless to the skin. Many factors have to be considered in the selection of tissue compensation materials for 3D printing. It is necessary and urgent to develop a new bolus material with combined advantages of easy-to-fabricate, convenient-to-use, low-cost, good-fit to skin contour, and antibacterial properties.

### Manufacturing Process

**Figure 1:**
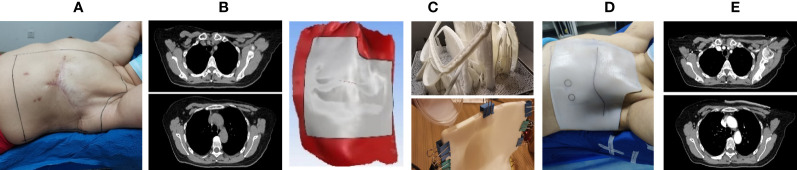
The schematic of the manufacturing process of a three-dimensional (3D) bolus. **(A)** Patients were positioned in standards and their body contour was marked; **(B, C)** 3D-modeling software was used to create a patient-specific bolus based on CT images and marks; **(D)** the fitness of the 3D-printed bolus was verified; **(E)** a radiotherapy plan which referenced the second CT scan with the 3D bolus in the treatment planning system (TPS) was formulated.

The production process of the 3D-printed bolus is basically similar in many studies, (shown in **Figure 1**) (26, 30, 34, 36, 37, 41). The approximate desired radiotherapy bolus area is marked on the anthropomorphic phantom or patient. The external contour is created from the CT scan, which is then expanded by the desired thickness of the bolus and subtracted from the expansion. Then the material is cut down to cover only the treatment area as defined during simulation. The result is the geometry of the desired bolus as a contour. This contour can then be converted into an STL file which can be processed into instructions for the 3D printer and the designed bolus is printed out. What needs special attention is that it is difficult to 3D print a perfectly solid piece of plastic (it tends to deform). Most prints are done with 2-3 shell layers on the outer edges of the object with some sort of lattice or honeycomb structure on the inside. The higher infill means having a more densely packed lattice, which leads to a more homogeneous bolus that could improve dose distribution on the patients’ skin. Subsequently, a radiotherapy plan which references a new CT scan with the 3D bolus is made in the treatment planning system (TPS) after the fitting degree is verified to be satisfactory (25, 26, 28, 29, 39, 40, 42).

## Pre-Clinical Evidence

During the pre-clinical study of Shin-Wook Kim and Jae Won Park, it was found that the difference between the calculated dose and the measured dose under the bolus was less than 1%, and the 3D-printed bolus improved the prescription dose metrics: Dmean (mean dose), Dmin (minimum dose), D90% (dose covered 90% of the target volume), and V90% (volume received 90% of the prescribed dose) (36, 39, 42) (Data comparison is shown in **Table 2**).

**Table 2:** Pre-clinical dosimetric evaluation of the 3D-printed bolus.

Authors	Lesion	Dosimetric evaluation
Kim et al. (36)	Nose	Dmax: 101.3% *vs.* 101.3% (without bolus *vs.* 3D bolus). Dmin: 25.4% *vs.* 90.0% (without bolus *vs.* 3D bolus). Dmean: 86.4% *vs.* 95.5% (without bolus *vs.* 3D bolus). D90%: 62.7% *vs.* 91.6% (without bolus *vs.* 3D bolus). V90%: 53.5% *vs.* 100% (without bolus *vs.* 3D bolus).
Park et al. (39)	Head	Dmax: 107.7% *vs.* 107.7% (without bolus *vs.* 3D bolus). Dmin: 14.2% *vs.* 86.3% (without bolus *vs.* 3D bolus). Dmean: 85.7% *vs.* 102.3% (without bolus *vs.* 3D bolus). V95%: 50.8% *vs.* 86.3% (without bolus *vs.* 3D bolus). V90%: 57.8% *vs.* 99% (without bolus *vs.* 3D bolus)
Zhang et al. (42)	Perineum	Dmax: 182.9 cGy *vs.* 118.9 cGy *vs.* 114.8 cGy (without bolus *vs.* commercial bolus *vs.* 3D bolus). Dmean: 146.2 cGy *vs.*107.7 cGy *vs.* 104.1 cGy (without bolus *vs.* commercial bolus *vs.* 3D bolus). HI: 0.56 *vs.* 0.15 *vs.* 0.11 (without bolus *vs.* commercial bolus *vs.* 3D bolus).
Canters et al. (20)	Skin	GTV coverage (V95%): 84% *vs.* 97% (commercial bolus *vs.* 3D bolus). CTV coverage (V85%): 88% *vs.* 97% (commercial bolus *vs.* 3D bolus).
Baltz et al. (23)	Head	Maximum air gap: 4 mm. Mean (± SD) dose: 206± 2.7 cGy. Average error: 2.4%.
Park (29)	Breast	Average difference: -5.1% *vs.* -0.5% (3 mm commercial bolus *vs.* 3 mm PLA bolus), -3.2% *vs.* -1.1% (5 mm commercial bolus *vs.* 5 mm PLA bolus).
Su et al. (33)	Head	OAR-eye: 41.7% *vs.* 23.1% (commercial bolus *vs.* 3D bolus). OAR-lens: 83.3% *vs.* 62.2% (commercial bolus *vs.* 3D bolus).
Ricotti et al. (35)	Phantom	Maximum dose difference: 3.3% *vs.* 8.2% (3D-ABS bolus *vs.* 3D-PLA bolus). Dmax shift: (10% infill) 12 mm *vs.* 12 mm (3D-ABS bolus *vs.* 3D-PLA bolus). (20% infill) 11 mm *vs.* 12 mm (3D-ABS bolus *vs.* 3D-PLA bolus). (40% infill) 8 mm *vs.* 8 mm (3D-ABS bolus *vs.* 3D-PLA bolus). (60% infill) 2 mm *vs.* 0 mm (3D-ABS bolus *vs.* 3D-PLA bolus)
Hou et al. (32)	Head	PTV coverage: 98.3% *vs.* 100% (commercial bolus *vs.* 3D bolus)
Park et al. (26)	Head	Dose of surface: 88.3-100.2 cGy *vs.*88.3-98.9 cGy (3D-Silicon bolus *vs.* 3D-Clear Flex 30 bolus). %diff: (scalp) −1.22% *vs.* −4.5% (3D-Silicon bolus *vs.* 3D-Clear Flex 30 bolus). (ear) −0.96% *vs.* −4.7% (3D-Silicon bolus *vs.* 3D-Clear Flex 30 bolus). (nose) −1.85% *vs.* −0.85% (3D-Silicon bolus *vs.* 3D-Clear Flex 30 bolus). (chin) −1.74% *vs.* −5.16% (3D-Silicon bolus *vs.* 3D-Clear Flex 30 bolus).

Dmax, maximum dose of the target volume; Dmin, minimum dose of the target volume; Dmean, mean dose of the target volume; D90%, the dose that covers 90% of the target volume; V90%, the target volume that receives over the 90% of the prescribed dose; HI, dose homogeneity index; GTV, gross tumor volume; CTV, clinical target volume; PTV, planning target volume; OAR, organs at risk; %diff(the percentage difference), differences between calculated and measured doses at the surface were acquired to investigate the uncertainty of bolus structures. %diff=100x(measured dose-calculated dose)/calculated dose.

The 3D-printed bolus could better ensure the coverage of the target area and the accuracy of radiotherapy dose because of good adhesion (maximum air gap less than 4 mm) (23, 33). Richard A. Canters found that a 3D bolus leads to an improvement of GTV coverage (V95%) from 84% to 97% (p=0.05); CTV coverage (V85%) improved from 88% to 97% (p=0.006) (20). The new material hydrogel or silicon bolus significantly increased the dose in the dose volume histogram (DVH). A 3D bolus gives 100% of PTV coverage at the dose level of 90%, while a conventional bolus with more air gap had only 98.3% (26, 32). So-Yeon Park compared the 3D-printed bolus and commercial bolus in postmastectomy radiotherapy. The theoretical and practical dose errors in 200 and 300 cc volume with the 3D bolus were not remarkable, which were -0.7% and -0.6% for 3 mm, -1.1% and -1.1% for 5 mm (no statistically significant difference), respectively, however, the dose errors of the commercial bolus were -5.1% and -3.2% for 3 mm, -6.3% and -4.2% for 5 mm with a statistically significant difference (P<0.001), indicating that the 3D-printed bolus could not only reduce the daily positioning error, but also overcome the dose reduction caused by the air gap between the bolus and skin surface (29).

## Clinical Evidence

### Dosimetry Evaluation

It is critical to ensure that the skin surface receives the adequate prescribed radiation dose in the treatment of superficial tumors. Martin J. Butson et al. showed that the air gaps under the bolus would reduce skin surface dose as measured by the Attix parallel plate ionization chamber and radiochromic film detection. An unwanted air gap of 4-10 mm would decrease the superficial dose by about 4-10% (43). After preclinical explorations, 3D-printed boluses have overcome the disadvantages of traditionally available boluses by not only reducing air gaps in order to achieve doses closer to a uniform prescription, but also protecting organs at risk. Therefore, the research on the clinical applications of 3D-printed boluses are being gradually developed (**Table 3**).

**Table 3:** Clinical dosimetric evaluation of the 3D-printed bolus.

Authors	Disease	Patients	Thickness	Prescribe dose	Dosimetric evaluation
Robar et al. (25)	Breast cancer	16	5 mm	5000 cGy	Air gap: 5 ± 3 mm *vs.* 3 ± 3 mm (commercial bolus *vs.* 3D bolus)
Baltz et al. (23)	Head and neck cancer	1	–	5000 cGy	Air gap: 4 mm (max). %diff: 5.3%. Measurement average dose: 204 cGy, within ±10 cGy of TPS.
Canters et al. (20)	Non melanoma skin cancer	26	–	4800-6000 cGy	GTV coverage (V95%): 87.6% *vs.* 85.3% (theory dose on TPS *vs.* 3D bolus). CTV coverage (V85%): 96.4% *vs.* 92.7% (theory dose on TPS *vs.* 3D bolus).
J.W. Park et al. (37)	Kimura’s disease (Auricle)	1	5 mm	4000 cGy	Dmax: 2160 cGy *vs.* 2120 cGy *vs.* 2120 cGy (without bolus *vs.* commercial bolus *vs.* 3D bolus). Dmean: 1930 cGy *vs.* 1960 cGy *vs.* 1960 Gy (without bolus *vs.* commercial bolus *vs.* 3D bolus). V95%: 70.2% *vs.* 93.7% *vs.* 92% (without bolus *vs.* commercial bolus *vs.* 3D bolus)
Hou et al. (28)	Breast cancer	2	–	5000 cGy	Dmax: 5483-5508 cGy *vs.* 5523-5538 cGy (theory dose on TPS *vs.* 3D bolus). Dmin: 3298-3332 cGy *vs.* 2784-2932 cGy (theory dose on TPS *vs.*3D bolus). HI: 0.09-0.11 *vs.* 0.10-0.12 (theory dose on TPS *vs.* 3D bolus).
Park et al. (30)	Breast cancer	6	2 mm	5040 cGy	Differences to prescribed dose: 6% *vs.* 3% (commercial bolus *vs.* 3D bolus). %diff to 180 cGy: 4.43% *vs.* 0.47% (commercial bolus *vs.* 3D bolus). 3D bolus group: the radiation dose for OARs reduced by 20%.
Chiu et al. (40)	Head and neck cancer	7	1-3 mm	2000-3000 cGy	Differences to prescribed dose: Within 5%. Dose of nose: 200 cGy *vs.* 190.5-200.2 cGy (theory dose on TPS *vs.* 3D bolus). In-vivo measurement deviation: -5.12% to 0.09%
Zhang et al. (42)	Paget’s disease	10	–	5000-7000 cGy	HI: 0.03-0.15 (median of 0.06).
Lukowiak et al. (44)	Basal cell carcinoma (eye)	11	–	6000 cGy	MLI: 28.2-99% *vs.* 92.5-98.4% (commercial bolus *vs.* 3D bolus). Differences to prescribed dose: (mean) 24% *vs.* 5% (commercial bolus *vs.* 3D bolus). (max) 8% *vs.* 2.5% (commercial bolus *vs.* 3D bolus)
Su et al. (33)	Rhabdomyosarcoma	1	10 mm	5040 cGy	OAR-left kidney: 4586.7 cGy *vs.* 2834.5 cGy (commercial bolus *vs.* 3D bolus).
Zhao et al. (38)	Head and neck disease	4	10 mm	6000 Gy	Air gaps: 11 mm *vs.* 2-4 mm (commercial bolus *vs.* 3D bolus). Dmax: 118.8-120.2% *vs.* 106.7-118.5% (commercial bolus *vs.* 3D bolus). Dmean: 94.7-104.7% *vs.* 100.5-102.3% (commercial bolus *vs.* 3D bolus). OAR-Brain: Dmax: 103.8% *vs.* 82.7% (commercial bolus *vs.* 3D bolus). Dmean: 7.2% *vs.* 3.8% (commercial bolus *vs.* 3D bolus). OAR-eyes: Left-Dmean: 101.5% *vs.* 91.5% (commercial bolus *vs.* 3D bolus). Right-Dmean: 93.2% *vs.* 72.2% (commercial bolus *vs.* 3D bolus). OAR-optic nerves: Left-Dmean: 94.7% *vs.* 63.7% (commercial bolus *vs.* 3D bolus). Right-Dmean: 84.9% *vs.* 44.5% (commercial bolus *vs.* 3D bolus).

TPS, treatment planning system; MLD (matching level index), measure degree to which 3D-printed or commercial boluses were accurately mapped to the reference one in the TPS, range from 0 to 100%, where 100% indicates a perfect fit for the 3D-printed or commercial bolus to the reference one. ML= (V1/Vr)/(V1/V2)*100%, where V1 is the volume of the 3D-printed or commercial bolus contained in the volume of the reference bolus; Vr represents the total volume of the reference bolus; and V2 is the volume of the 3D-printed or paraffin bolus.

James L. Robar et al. applied a 3D-printed bolus in 16 patients with postmastectomy radiotherapy. Results found that the patient surface fit accuracy of the 3D-printed bolus was significantly improved, air gaps of more than 5 mm were decreased from 30% to 13% (P<0.0003), and the maximum air gaps diminished from 5 ± 3 mm to 3 ± 3 mm (25). Another application of the 3D-printed bolus in head and neck disease found that the maximum subcutaneous air gap was only 4 mm and the actual irradiation dose on the skin surface was only ±10 cGy different from the planned dose (23, 37).

The 3D-printed bolus has a significant increase in skin fit compared to the commercial bolus, thus ensuring adequate irradiation dose. Richard A. Canters, etc. compared dose coverage differences between a traditionally generated bolus and 3D-printed silicon bolus among 11 non-melanoma skin cancer cases, and found that GTV (V95%) rose from 84% to 97% (p = 0.05), CTV (V85%) rose from 88% to 97% (p = 0.006), and emphasized the superior advantage of 3D-printed bolus dosimetry distribution (20). Two other studies found that both 3D-printed silica gel (3D-SRB) or 3D-printed PLA (3D-PLA) was closer to the prescribed dose (0.47% *vs.* 4.43%), improved dose uniformity by 45%, and improved the precision of the dose absorbed by the chest wall to 3% (28, 30). Tsuicheng Chiu and Zhang Min, etc. demonstrated that a cast silicon bolus showed high homogeneity and exceptional fit to patients and enabled predictable and reproducible dosimetry (40, 42). Magdalena Lukowiak et al. made a bolus for 11 patients with basal cell carcinoma of the eye, and found that the fit between the reference bolus (planned in TPS) and the 3D-printed bolus was higher than that of the artificial paraffin bolus (92.5-98.4% *vs.* 28.2-99%). The minimum dose difference between the actual dose and the reference dose was <5%, maximum dose difference <2.5%, while the artificial paraffin bolus was as high as 24% and 8%, which emphasized the advantages of the adhesion and dose homogeneity of the 3D-printed bolus (44).

The 3D-printed bolus ensured the radiation dose in the target area. Kwangwoo Park found the continuous shape of the 3D bolus showed less relatively hot and cold spots, which resulted in improving dose conformity and uniformity, and the radiation dose for organs at risk (OARs) reduced by 20%, including lung dose decreases ranging from 24.5% to 40.5%, significantly reducing the risk of radiation pneumonia (30). Su et al. pointed out that a modulation of electron radiation therapy (MERT)-optimized 3D bolus with the distal contour of PTV is less variable compared with a commercial bolus, which will reduce areas of unwanted dose enhancement and protect organs at risk. In the treatment planning for a pediatric patient with a rhabdomyosarcoma adjacent to the left kidney, a 38.2% mean dose reduction to the kidney was obtained compared with a uniform bolus (33). Yizhou Zhao et al. pointed out that the 3D-printed bolus enhanced both patient comfort and reproducibility; ensuring the target dose, at the same time protecting the OAR. Making this technique usable in a wide variety of radiotherapy treatments for superficial tumors (38).

### Radiation Dermatitis

Skin injury is one of the most common toxicities and side effects in radiotherapy. Although most skin injuries after radiotherapy are relatively mild and reversible, there are still great differences in severity and prognosis with the increase of dose (45). Acute radiation dermatitis usually occurs within 90 days after radiation exposure. The main symptoms are erythema, moist desquamation, and even ulceration. The severity is closely related to the dose. It is easier to cause skin fold moist desquamation when the cumulative dose meets or exceeds 4000 cGy. The most severe reactions usually occur in the 1-2 weeks after completion of radiotherapy treatment (46, 47). However, the incubation period of chronic radiation dermatitis is months or years, mainly manifested as skin fibrosis, atrophy, and malignant changes, etc. It usually presents as mild acute skin reactions or normal skin, and is generally difficult to self-repair (45).

Both the traditional bolus and 3D-printed bolus not only increase the irradiation dose on the skin surface, but also aggravate the skin reaction to varying degrees (48). A study reported by Nancy Lee et al. (49) observed five cases of head and neck tumor patients treated with a commercial bolus, and the average dose increased by 18% after the use of the bolus. All patients had acute skin reactions, including one case of grade 3 toxicity (RTOG classification standard). Minh Thi Tieu, etc. recorded 254 patients who received postmastectomy radiotherapy. The incidence of acute skin reaction was 12% (bolus group) and 2.7% (without bolus group), and the whole chest wall using the bolus was associated with a significant interruption of treatment (P=0.012), discontinued treatment, and further improved the local recurrence rate (HR=4.8) of the skin (50). A subsequent similar study also found that the rate of level 3 toxicity was as high as 47% for daily use of a bolus versus only 26% for once every other day use (95%CI 2.05-6.26, P<0.001). In addition to the effect of cumulative dose on the skin surface, smoking history (95%CI 1.09-8.01, P=0.03) and radiation energy (95%CI 0.23-0.97, P=0.04) were all correlated (51).

At present, the clinical application of the 3D-printed bolus is limited. There is no large-scale multi-center, long-term follow-up, prospective study to explore the radiation dermatitis when using a 3D-printed bolus. The observation of the incidence of skin toxicity and side effects is still lacking the support of abundant clinical data (52, 53).

#### Cost-Effectiveness

The production process of 3D printing a bolus is becoming more and more mature. Compared with a conventional tissue bolus, the cost consumption is mainly reflected in the material cost and labor, and the cost and time loss caused by the production tool or printer cost, equipment maintenance, and printing failure can be ignored (details in **Table 4**) (23, 41). Based on this, Christine Albantow et al. compared 24 cases of a 3D-printed nose bolus made of paraffin and PLA. The time cost and average material cost of the paraffin bolus were $138.54 and $20.49, respectively, while the 3D printing bolus costs were only $10.58 and $13.87, respectively, indicating that the 3D-printed bolus was more cost-effective (22). Although a 3D-printed bolus could significantly reduce labor and material costs, there are still significant differences between different materials and labor (20, 29, 41, 44, 54). In addition, James L. Robar et al. considered the placement time of a bolus in radiotherapy and found that the installation time of the 3D-printed bolus decreased from 104 s to 76 s compared with the traditional bolus, greatly reducing the overall time cost (25). Tsuicheng Chiu et al. and Richard A. Canters et al. optimized the production method and medical treatment process of the 3D-printed bolus, so as to reduce the frequency of patient visits and artificial labor, reduce the waiting time and material cost, and provide patients with lower price and a more comfortable personalized 3D-printed bolus (20, 40).

**Table 4:** Cost-effectiveness of the 3D-printed bolus.

Authors	Material	Cost	Print time/hours
Canters et al. (20)	PLA	$33.2/print and material	7 (range 5–10)
Christine Albantow et al. (22)	PLA	$33.81-45.36/labor(hour)	8.8-12.4
So-Yeon Park (29)	PLA	$30/material	12
W. Zou et al. (41)	PLA	Depend on the printing size	6-9
James L. Robar et al. (25)	PLA	$10/material	–
Magdalena Lukowiak et al. (44)	PLA	$10.58(staff time), $13.87(materials)	–
Kwangwoo Park et al. (30)	PLA	–	6
Hou Yanjie et al. (28)	Silicon, PLA	–	8.5-12
Jong Min Park et al. (26)	Silicon	–	3-32
Tsuicheng Chiu (40)	Silicon	$7.75-$28.41/material	4-6
Sarah Burleson et al. (34)	ABS, PLA	$3,000/material and labor	4-6
Shin-Wook Kim et al. (36)	ABS-M30	–	3-4.5
Garrett C. Baltz et al. (23)	Agilus‐60	$2,381.50/material and labor	40

## Discussion

The advent of the 3D-printed bolus has been revolutionary in superficial tumor radiotherapy. Regarding the current trend of the development of custom 3D-printed boluses, an increasing number of available reports have shown that this approach can reduce the air gap, improve the accuracy and uniformity of dose, better protect normal tissues, and has clear advantages in cost and time efficiencies. However, the clinical evidence is not robust and certain questions remain unanswered. There is not yet a consensus about the material choice, and frequency of application. With the use of any bolus there is a likelihood of adverse skin reactions. There is a need for further study to make the best use of the advantages of 3D-printed boluses while reducing acute skin reactions.

## Author Contributions

These authors named XRW and XTW who wrote the manuscript have contributed equally to this work. ZX, YZ, FL, and BS were responsible for guiding the writing of the paper. JM, SY, and TH were responsible for the collection of literature materials. LL was responsible for the overall revision. All authors contributed to the article and approved the submitted version.

## Conflict of Interest

The authors declare that the research was conducted in the absence of any commercial or financial relationships that could be construed as a potential conflict of interest.

## Publisher’s Note

All claims expressed in this article are solely those of the authors and do not necessarily represent those of their affiliated organizations, or those of the publisher, the editors and the reviewers. Any product that may be evaluated in this article, or claim that may be made by its manufacturer, is not guaranteed or endorsed by the publisher.
